# Development of an ELISA-array for simultaneous detection of five encephalitis viruses

**DOI:** 10.1186/1743-422X-9-56

**Published:** 2012-02-27

**Authors:** Xiaoping Kang, Yuchang Li, Li Fan, Fang Lin, Jingjing Wei, Xiaolei Zhu, Yi Hu, Jing Li, Guohui Chang, Qingyu Zhu, Hong Liu, Yinhui Yang

**Affiliations:** 1 Department of Virology, State Key Laboratory of Pathogen and Biosecurity, Beijing Institute of Microbiology and Epidemiology, Beijing 100071, China

## Abstract

Japanese encephalitis virus(JEV), tick-borne encephalitis virus(TBEV), and eastern equine encephalitis virus (EEEV) can cause symptoms of encephalitis. Establishment of accurate and easy methods by which to detect these viruses is essential for the prevention and treatment of associated infectious diseases. Currently, there are still no multiple antigen detection methods available clinically. An ELISA-array, which detects multiple antigens, is easy to handle, and inexpensive, has enormous potential in pathogen detection. An ELISA-array method for the simultaneous detection of five encephalitis viruses was developed in this study. Seven monoclonal antibodies against five encephalitis-associated viruses were prepared and used for development of the ELISA-array. The ELISA-array assay is based on a "sandwich" ELISA format and consists of viral antibodies printed directly on 96-well microtiter plates, allowing for direct detection of 5 viruses. The developed ELISA-array proved to have similar specificity and higher sensitivity compared with the conventional ELISAs. This method was validated by different viral cultures and three chicken eggs inoculated with infected patient serum. The results demonstrated that the developed ELISA-array is sensitive and easy to use, which would have potential for clinical use.

## Background

Japanese encephalitis virus(JEV), tick-borne encephalitis virus(TBEV), eastern equine encephalitis virus (EEEV), sindbis virus(SV), and dengue virus(DV) are arboviruses and cause symptoms of encephalitis, with a wide range of severity and fatality rates [1]. Establishment of an accurate and easy method for detection of these viruses is essential for the prevention and treatment of associated infectious diseases. Currently, ELISA and IFA are the methods which are clinically-available for the detection of encephalitis viral antigens, but they could only detect one pathogen in one assay [2,3].

There are a variety of different methods available for identifying multiple antigens in one sample simultaneously, such as two-dimensional gel electrophoresis (2-DE), protein chip, mass spectrometry, and suspension array technology [4-6]. However, the application of these techniques on pathogen detection is still in an early phase, perhaps due to the complicated use and high cost.

Antibody arrays for simultaneous multiple antigen quantification are considered the most accurate methods [7-10]. Liew [11] validated one multiplex ELISA for the detection of 9 antigens; Anderson [12] used microarray ELISA for multiplex detection of antibodies to tumor antigens in breast cancer, and demonstrated that ELISA-based array assays had the broadest dynamic range and lowest sample volume requirements compared with the other assays.

However, the application of ELISA-based arrays is currently limited to detection of cancer markers or interleukins; no detection of pathogens has been reported. In this study, we developed an ELISA-based array for the simultaneous detection of five encephalitis viruses. Seven specific monoclonal antibodies were prepared against five encephalitis viruses and used to establish an ELISA-array assay. The assay was validated using cultured viruses and inoculated chicken eggs with patient sera. The results demonstrated that this method combined the advantage of ELISA and protein array (multiplex and ease of use) and has potential for the identification of clinical encephalitis virus.

## Methods

### Monoclonal antibody preparation

Monoclonal antibodies were prepared from hybridoma cell lines constructed by Prof. Zhu et al. Purification was conducted by immunoaffinity chromatography on protein G affinity sepharose [13]. Specific monoclonal antibodies (4D5 against JEV, 2B5 against TBEV, 1F1 against SV, 2B8 against serotype 2 DV, 4F9 against serotype 4 DV, 4E11 against EEEV, and 2A10 against *Flavivirus*) were selected for this study. All of the antibodies were raised according to standard procedures.

Using 4D5, 2B5, 1F1, 2B8, 4F9, and 4E11 as capture antibodies, detection antibodies (2A10, 1 F1, and 4E11) were coupled to biotin-NHS ester(Pierce, Germany) at 4°C for 3 h according to the manufacturer's instructions. Unincorporated biotin was removed by Desalt spin column (Pierce). Immunologic reactions were reported by Streptavidin-HRP (CWBIO, Beijing, China) and Super Signal ELISA Femto Maximum sensitive substrate. Purified goat-anti mouse antibody was used as a positive control.

### Virus culture

JEV and DV were cultured in C6/36 cells; SV, TBEV, and EEEV were cultured in BHK-21 cells. The culture of TBEV and EEEV was conducted in biosafety level 3 facility, however, JEV, DV and SV were conducted in biosafety level 2 facility. Viral titers were determined by the 50% tissue culture infectious dose (TCID_50_) method. All the cultures were inactivated by 0.025% β-propionolactone at 4°C overnight, then 37°C for 1 h to decompose β-propionolactone.

### Antibody spotting and optimization

Antibodies were spotted using a BIODOT machine (BD6000;California, USA) on ELISA plates (30 nl/dot). The plates were blocked with 3% BSA-PBS in 37°C for 1 h, followed by washing 3 times with PBS containing 0.1% Tween-20 for 2 min each. Then, the plates were dried, sealed, and stored at 4°C before use [11].

When spotting, different spotting buffers and concentrations of capture monoclonal antibodies were evaluated to optimize the ELISA-array assay. The optimization was evaluated by dot morphology and signal intensity. The tested spotting buffers included 1 × phosphate buffer saline (PBS), PBS +20% glycerol, and 1 × PBS + 20% glycerol+0.004% Triton-X100. A range of monoclonal antibody concentrations (0.0125, 0.025, 0.05, 0.1, and 0.2 mg/ml) were compared.

Following a double antibody sandwich format, printed plates were incubated sequentially with inactivated viral cultures, biotin-labeled detecting antibody, HPR-labeled avidin, and substrate, followed by signal evaluation.

### ELISA-array analysis

Antigen binding was performed in PBS(containing 0.1% Tween-20 and 5% FCS) at 37°C for 2 h, followed by washing 3 times(1 × PBS containing 0.1% Tween-20). Incubation of ELISA plates with biotinylated detecting antibody cocktails was performed in PBS (containing 0.1% Tween-20 and 5% FCS) at 37°C for 2 h. After washing, specific binding of the detecting antibodies was reported by streptavidin-HRP and stained with Super Signal ELISA Femto Maximum sensitive substrate (Thermo scientific, Rockford, USA) [11,14,15]. Visualization of the plate was performed in AE 1000 cool CCD image analyzer(Beijing BGI GBI Biotech Company., LTD, China). The signal intensity and background of each spot was read out and recorded with "Monster"software. The positive signals were defined as a signal value > 400 and a signal value (sample)/signal value (negative) > 2.

### Conventional ELISA

The identical antibodies used in the ELISA-array format were also tested in a conventional ELISA format to determine the difference in sensitivity and specificity of the two methods. The conventional ELISAs were performed at the same time as the ELISA-array assays to ensure similar reaction conditions. The conventional ELISAs were performed in an identical maner to the ELISA-array, except that antibodies were coated at a concentration of 2 μg/mL in PBS (pH 7.4), and substrate TMB was used instead of Super Signal ELISA Femto Maximum sensitive substrate [16,17].

### Clinical sample treatment and chicken egg inoculation

Three serum samples were collected from patients with nervous system symptoms and histories of tick bites. The serum samples were treated with penicillin and streptomycin, then inoculated into the allantoic cavities of chicken eggs. 3 days later, the liquid was collected and divided into two portions (one for inactivation and one for RNA extraction). The RNA and inactivated samples were stored at -70°C before use.

### Real-time RT-PCR assay

RNA was extracted from the inoculated chicken eggs using a RNeasy mini kit (Qiagen Inc., Valencia, CA, USA) according to the manufacturer's instructions. All RNA extraction procedures were conducted at BSL-3 facilities. The primers and probes were used as previously described [18]. The real-time RT-PCR was conducted with a Quti-teck q-RT-PCR Kit (Qiagen Inc,). The reaction consisted of 10 μL of 2 × reaction buffer(0.2 μL reverse transcription enzyme, and 250 nmol/l primers and probes). RNA and deionized water were added to a final volume of 20 μl. PCR was performed with a LightCycler 2.0 (Roche, Switzerland) [19].

## Results and discussion

### Optimization of the ELISA-array assay

**Figure 1:**
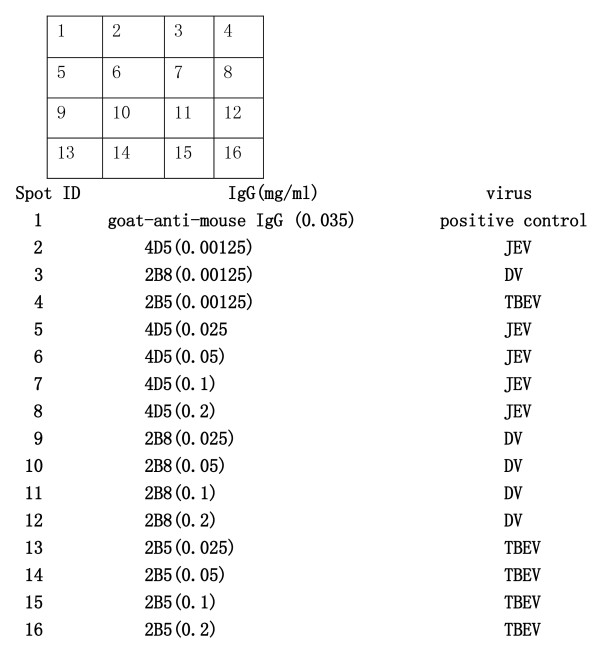
**Print layout of antibodies spotted on ELISA plates, spot volume = 30 nl**.

The spotted array layout is depicted in Figure 1 and the efficacy of three different spotting buffers on the quality of the printed ELISA-arrays were investigated by spot morphology observation and signal intensity comparison.

**Figure 2:**
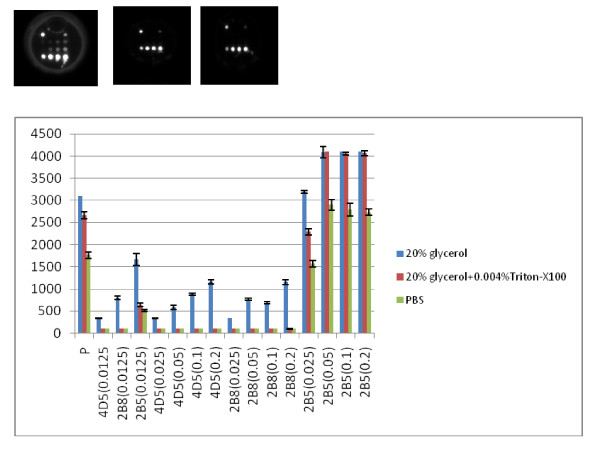
**Evaluation of different spotting buffers and antibody concentrations in ELISA-array application**. **a **spot morphology visualization by using 20% glycerol-PBS buffer; **b **spot morphology visualization by using PBS + 20% glycerol+0.004%Triton-X100 buffer; **c **spot morphology visualization by using PBS buffer. **d **Signal intensity comparisons of different solutions and spotting concentration of 2B5.

The spotting concentration of the capture antibodies varied from 0.2 to 0.0125 mg/ml (each was serially diluted 2-fold). The efficacy of the spotting concentration of the capture antibodies was evaluated by virus culture detection, the proper spotting concentration was determined by a combination of minimized cross reaction and higher signal intensity. Figure 1 illustrates the array layout and Figure 2 demonstrates the result of the three spotting buffers and spot concentration of antibody 2B5 by TBE virus culture detection. Cross reaction detection was also conducted by applying JEV, YF, and DV cultures.

Spot morphology observation (Figures 2a, b, and 2c) demonstrated that spotting buffer containing PBS with 20% glycerol produced tailed spot morphology; buffers containing PBS alone and PBS with 20% glycerol+0.004% Triton-X100 gave good spot morphology (round and full). Buffers containing PBS with 20% glycerol and PBS with 20% glycerol+0.004% Triton-X100 produced higher signal intensities than PBS alone. Thus, PBS with 20% glycerol+0.004% Triton-X100 was adopted as the optimized spotting buffer for subsequent experiments. Simultaneously, the spot concentration evaluation suggested that 0.05 mg/ml was optimal. At this concentration, the signal intensity was higher and the cross-reaction did not appear (Figure 2d). Consequently, spotting concentration optimization of other capture antibodies (4D5, 1F1, 4E11, and 2B8) demonstrated that 0.05 mg/ml was also suitable(data not shown).

**Figure 3:**
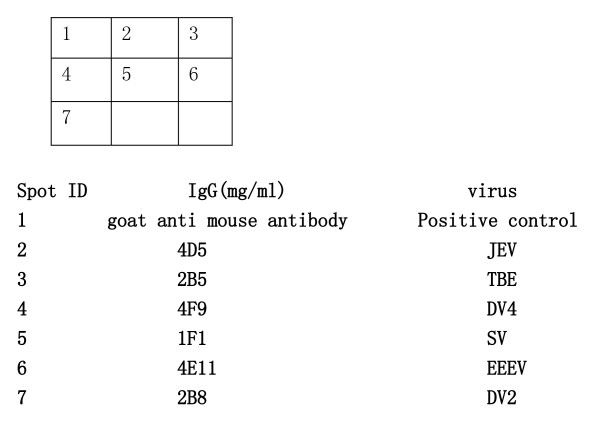
**the optimized array layout, spot volume = 30 nl**.

The optimized ELISA array layout is shown in Figure 3, which was applied in the following experiments.

### Sensitivity and specificity of ELISA-array compared with conventional ELISAs

Successful detection of viral pathogens requires a test with high sensitivity and specificity. To evaluate the performance of the designed antibody arrays, the specificity and sensitivity of the individual analytes were examined. By testing serially-diluted viral cultures, including DV-2, DV-4, JEV, TBE, SV, and EEEV, the sensitivity of ELISA-array and the identical conventional ELISA were compared (Table 1). The detection limit of the two methods was compared and demonstrated. The cross-reactivity test was conducted using BHK-21 and vero cell lysate, Yellow fever virus (YFV) cultures (5 × 10^5 ^TCID_50_/ml, West Nile virus(WNV) cultures(2 × 10^6 ^TCID_50_/ml), and Western equine encephalitis virus(1 × 10^7 ^TCID_50_/ml). The results demonstrated that neither the ELISA-array nor traditional ELISA displayed cross-reactivity.

**Table 1:** Detection limit comparison between ELISA-array and traditional ELISA

	ELISA-array (TCID_50_/ml)	Traditional ELISA(TCID_50_/ml)
JEV	1.5 × 10^4^	6 × 10^4^
TBEV	8 × 10^3^	8 × 10^4^
DV2	6.25 × 10^4^	1.25 × 10^5^
EEEV	8 × 10^4^	1.6 × 10^5^
DV4	6 × 10^4^	6 × 10^5^
SV	2.5 × 10^6^	1.25 × 10^6^

### Single and multiplex detection of TBEV, JEV, DV, SV, and EEEV

**Figure 4:**
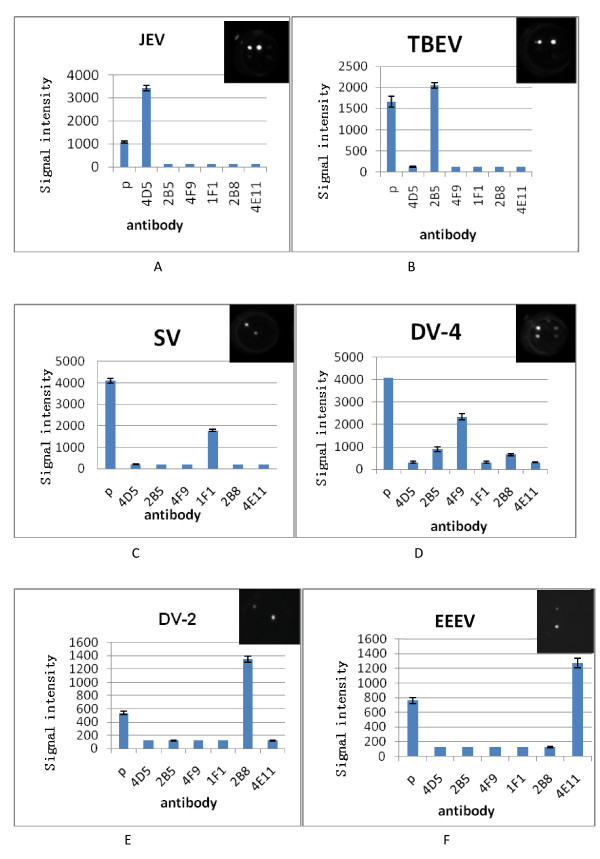
**The ELISA array detection results for single encephalitis virus**. **a**:Japanese encephalitis virus(JEV); **b**:Tick born encephalitis virus(TBEV); **c**:Sindbis virus(SV); **d**:Dengue-4 virus(DV-4);E:Dengue-2 virus(DV-2);F:Eastern equine encephalitis virus(EEEV).

**Figure 5:**
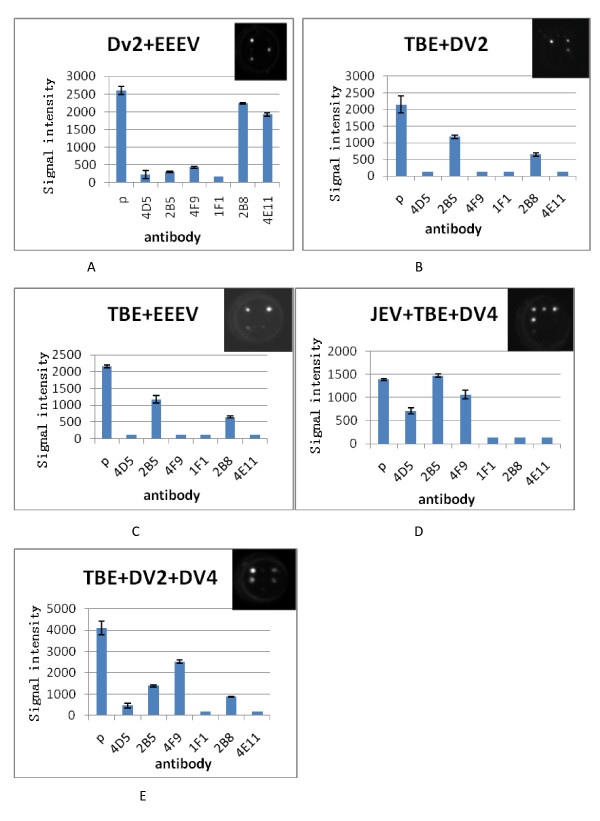
**The ELISA array detection results for multiplex encephalitis virus**. **a**: DV-2 and EEEV; **b**: TBEV and DV2; **c**:TBEV and EEEV; **d**: JEV, TBEV and DV-4; **e**: DV-2, DV-4 and TBEV.

Equal volumes of cultured TBEV, JEV, DV-2, DV-4, SV, and EEEV were prepared for single sample detection; two or three of the cultures were mixed for multiplex detection. A cocktail of biotin conjugated antibody (2A10, 4E11, and 1F1) was used in all tests. The results demonstrated that for all virus combinations, each virus was detected specifically, with no false-positive or-negative results (Figures 4 and 5).

### Validation by mock clinical samples

**Figure 6:**
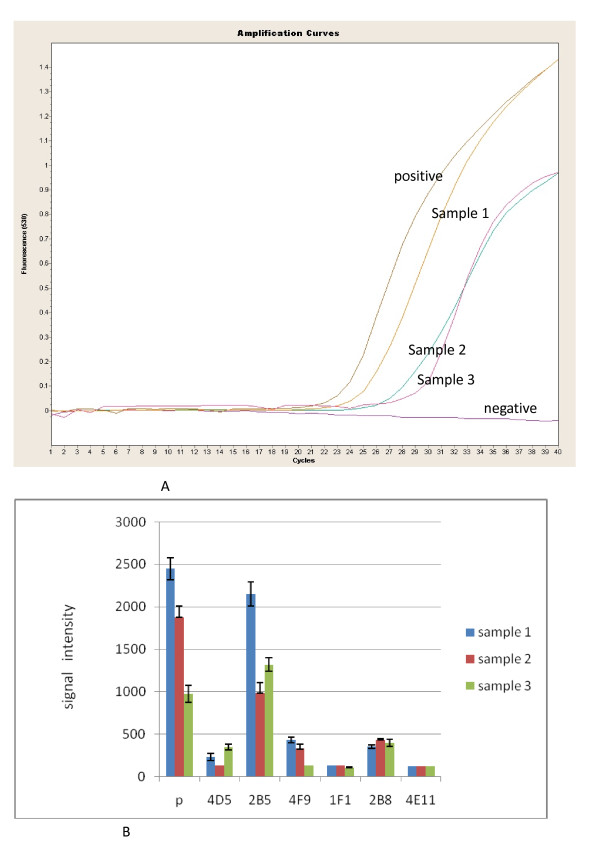
**Detection of three Chicken egg samples inoculated with infected human serum**. **a**: Real time RT-PCR assay result of the samples; **b**: ELISA Array result of the samples.

Chicken eggs inoculated with infected human serum were used for validation of the ELISA-array assay. All samples showed high reaction signals with capture antibody 2B5, which was specific for TBEV (Figure 6b). The ELISA-array assay suggested that the three patients were all infected with TBEV.

To verify the results tested by ELISA-array, RNA extracted from chicken eggs was applied to a real time-RT-PCR assay using primers and probes targeting TBEV. The results were also positive (Figure 6a). The consensus detection results confirmed that the ELISA-array assay was reliable.

## Discussion

To be widely used in the clinical setting, the detection system should be easy to use and can be performed by untrained staff with little laboratory and experimental experience. Moreover, when the volume of the clinical samples is limited and an increasing number of pathogens per sample needs to be tested, the detecting system should be high-throughput to allow detection of multiple pathogens simultaneously [6,20,21]. Multiple detection, easy to use, and affordability are requirements for detection methods in the clinical setting. Thus, an ELISA-array, which combines the advantages of ELISA and protein array, meets the above requirements.

It has been reported that an ELISA-array has been used in the diagnosis of cancer and auto-allergic disease [7,12]; however, No study has reported the detection of viral pathogens. In this study, we developed a multiplex ELISA-based method in a double-antibody sandwich format for the simultaneous detection of five encephalitis-associated viral pathogens.

The production of a reliable antibody chip for identification of microorganisms requires careful screening of capture of antibodies [14]. Cross-reactivity must be minimized and the affinity of the antibody is as important as the specificity. First, we prepared and screened 23 monoclonal antibodies against eight viruses and verified the specificity and affinity to the target viruses by an immunofluorescence assay. Then, the antibodies were screened by an ELISA-array with a double-antibody sandwich ELISA format. The antibodies which produced cross-reactivity and low-positive signals were excluded. Finally, six antibodies were selected as capture antibodies. Another monoclonal antibody, 2A10, which could specifically react with all viruses in the genus *Flavivirus *was used for detecting antibody against DV, JEV, and TBEV. For the detection of EEEV and SV, although the detecting and trapping antibodies were the same (1F1 and 4E11, respectively), the antibodies produced excellent positive signals. The epitope was not defined; however, we suspect that the antibodies both target the surface of the virions. As one virion exits as, many with the same epitope appear, thus no interference occurred using the same antibody in the double-antibody sandwich format assay.

Currently, the availability of antibodies suitable for an array format diagnostic assay is a major problem. In the ELISA-array assay, this problem exists as well. Because of the limitation of available antibodies, this assay could only detect 5 pathogens. In the future, with increasing numbers of suitable antibodies, especially specific antibodies against *Flavivirus*, this ELISA-array might be able to test more pathogens and be of greater potential use.

To make the assay more amenable to multiple virus detection, the assay protocol was optimized. In addition to the dotting buffer, the capture antibody concentration and the different virus inactivation methods (heating and β-propiolactone) were also compared and evaluated. Heat inactivation was performed by heating the viral cultures at 56°C for 1 h, and β-propiolactone inactivation was performed by adding β-propiolactone into the viral cultures to obtain a final concentration of 0.25‰, incubating at 4°C overnight, then decomposing β-propiolactone at 37°C for 1 h. The performance was evaluated based on the infectivity experiments and detecting the limit of virus titers (data not shown). The infectivity experiments demonstrated that both of the two methods could totally inactive the viruses, but the sensitivity limit assay suggested that virus treated by β-propiolactone retains better antigenicity than the heat-inactivation method. Thus, β-propiolactone treatment was chosen as the virus-inactivation method.

A conventional ELISA is a standard method in many diagnostic laboratories. We compared the ELISA-array with a conventional ELISA and confirmed that the advantage of the ELISA-array was evident with comparable specificity and higher sensitivity than ELISA. The time required for the ELISA-array is significantly less than for conventional ELISA (4 h vs. a minimum of 6 h, respectively). Furthermore, less IgG is required for printing than for coating ELISA plates. Coating of a single well in microtiter plate requires 100 μl of a 1 μg/ml antibody solution, which is equivalent to 100 ng of IgG. For the ELISA-array, only 30 nl of a 50 μg/ml antibody solution is required for each spot, which is equivalent to 1.5 ng of IgG. With the characteristics of ease of use, sensitivity, specificity, and accuracy, the ELISA-array assay would be widely accepted for clinical use.

## Competing interests

The authors declare that they have no competing interests.

## Authors' contributions

XK: designed the study, performed the laboratory testing, analyzed the test results, and co-wrote and edited the manuscript. H L and YL performed the virus cultures. LF performed laboratory testing. XC, FL, and GC performed real time-PCR. QZ and YY organized the overall project and helped edit the manuscript. All of the authors read and approved the final manuscript.

## Authors' information

State Key Laboratory of Pathogen and Biosecurity, Beijing Institute of Microbiology and Epidemiology, Beijing 100071, China
